# Machine Learning-Based Strength Prediction for Refractory High-Entropy Alloys of the Al-Cr-Nb-Ti-V-Zr System

**DOI:** 10.3390/ma14237213

**Published:** 2021-11-26

**Authors:** Denis Klimenko, Nikita Stepanov, Jia Li, Qihong Fang, Sergey Zherebtsov

**Affiliations:** 1 Laboratory of Bulk Nanostructured Materials, Belgorod State University, 308015 Belgorod, Russia; stepanov@bsu.edu.ru (N.S.); zherebtsov@bsu.edu.ru (S.Z.); 2 State Key Laboratory of Advanced Design and Manufacturing for Vehicle Body, College of Mechanical and Vehicle Engineering, Hunan University, Changsha 410082, China; lijia123@hnu.edu.cn (J.L.); fangqh1327@hnu.edu.cn (Q.F.)

**Keywords:** high-entropy alloys, machine learning, prediction, strength, structure

## Abstract

The aim of this work was to provide a guidance to the prediction and design of high-entropy alloys with good performance. New promising compositions of refractory high-entropy alloys with the desired phase composition and mechanical properties (yield strength) have been predicted using a combination of machine learning, phenomenological rules and CALPHAD modeling. The yield strength prediction in a wide range of temperatures (20–800 °C) was made using a surrogate model based on a support-vector machine algorithm. The yield strength at 20 °C and 600 °C was predicted quite precisely (the average prediction error was 11% and 13.5%, respectively) with a decrease in the precision to slightly higher than 20% at 800 °C. An Al_13_Cr_12_Nb_20_Ti_20_V_35_ alloy with an excellent combination of ductility and yield strength at 20 °C (16.6% and 1295 MPa, respectively) and at 800 °C (more 50% and 898 MPa, respectively) was produced based on the prediction.

## 1. Introduction

High entropy alloys (HEAs), which are sometimes also called multi-principal element alloys, were originally discovered by Yeh [1] and Cantor [2]. In contrast to traditional alloys, which are based on one principal element, HEAs are defined as alloys with five or more principal elements in equal or near-equal atomic percentage (5–35 at.%). HEAs have attracted great research interest [3,4,5,6] due to their high strength (including high-temperature strength), structural stability, hardness, and wear resistance, as well as good corrosion and oxidation resistance [3,4,5,6,7,8,9]. Their superior properties enable their application in a wide range of modern industries, for example, as high-temperature materials for future aerospace vehicles.

Promising candidates for a new generation of high-temperature materials are HEAs based on refractory elements (RHEAs). The first RHEA, consisted of several refractory elements (Mo, Nb, Ta, V and W), showed high strength up to 1600 °C but had high density (>12 g/cm^3^) [10,11]. At such high densities, the applicability of these alloys is significantly limited. Therefore, the attention was focused on the development of lighter alloys. Usage of lighter refractory elements made it possible to reduce the density of the alloys considerably. For example, the specific strength of a CrNbTiVZr alloy with a density of 6.57 g/cm^3^ was found to be higher than that for commercial nickel-based alloys [12,13] Further development of this approach can be associated with including low-density non-refractory elements (such as Al or Si) in RHEAs. Thus, modern RHEAs can contain a wider range of elements (Ti, Zr, Hf, V, Nb, Ta, Cr, Mo, W, Al, Co, Ni, Si) [3,14,15].

Basically, high-entropy alloys provide a vast compositional space for the design of new alloys. On the one hand, a huge compositional space provides an ampler opportunity to obtain alloys with improved properties. On the other hand, the development of new of alloys with desired properties by a conventional “trial and error” approach can be impractical. Several methods for an efficient search for new HEAs compositions have been suggested so far. Generally, they are focused on the prediction of either phase composition or mechanical properties. However, the phase composition obviously affects the properties of the alloys. Various approaches can be used to predict the constituted phases as a function of the chemical composition, including the phenomenological rules [16,17,18,19,20,21,22,23,24,25], calculation of phase diagrams (CALPHAD) approach [26,27,28,29,30], machine learning algorithms [31,32,33], and other computational methods such as ab initio, Monte-Carlo (MC) or molecular dynamics calculation (MD) [34,35,36,37]. Each of these approaches has its own strengths and weaknesses. For example, ab initio and MD are very time-consuming and can hardly be used to predict structures of the alloys in a high-throughput manner. Phenomenological rules are based on empirical observations and are easy for calculations, but the accuracy of their predictions does not exceed 72% [25]. CALPHAD modeling can be quite throughput, however it requires significant computing resources. In addition, the accuracy of the CALPHAD predictions can be limited due to the absence of a reliable database for HEAs [38]. The machine learning approach is sensitive to the size and composition of a training dataset, but the prediction accuracy of this method can attain more than 90% [32].

Prediction of properties of HEAs are mostly focused on (yield) strength or hardness. Since strong lattice distortion was initially considered as a specific feature of HEAs, great attention was paid both to the experimental and computational predictions of solid solution strengthening [39,40,41,42,43,44,45]. These models allow calculation of the yield strength with sufficient accuracy but can be applied for only alloys with a single-phase structure. In recent years, approaches based on machine learning algorithms were developed. In this case, mechanical properties can be predicted for both single-phase and multi-phase alloys with reasonable accuracy [46]. Wen et al. [47] in Al-Co-Cr-Cu-Fe-Ni system found 46 new alloys with hardness values higher than the best value in the training dataset by using the machine-learning approaches. Alloys with the highest hardness reported in the literature for the AlCoCrFeMnNi-based system was found by using a neural network [48]. Li et al. [49] used an approach based on a high-throughput simulation combined with machine learning to obtain medium entropy alloys with high strength and low cost. However, such calculations were aimed to room-temperature properties only. Articles devoted to the prediction of strength characteristics at elevated temperatures are very rare. Bhandari et al. [50] predicted yield strengths of MoNbTaTiW and HfMoNbTaTiZr at 800 °C and 1200 °C with high accuracy by using RF regressor model. Therefore, in this work, we have employed the machine learning method to predict mechanical properties of Al-Cr-Nb-Ti-V-Zr system RHEAs. Some of these alloys have already demonstrated high specific strength at T ≤ 800 °C in combination with reasonable ductility [51,52,53,54,55,56,57,58,59,60,61,62]. In addition to the machine learning prediction of the yield strength at room temperature, 600 °C and 800 °C, a combination of phenomenological criteria and CALPHAD modeling was used to screen alloys with a predominantly single-phase structure to ensure reasonable ductility. In addition, several Al-Cr-Nb-Ti-V-Zr alloys were produced and examined to evaluate the credibility of the predictions.

## 2. Materials and Methods

### 2.1. Computational Predictions

**Figure 1:**
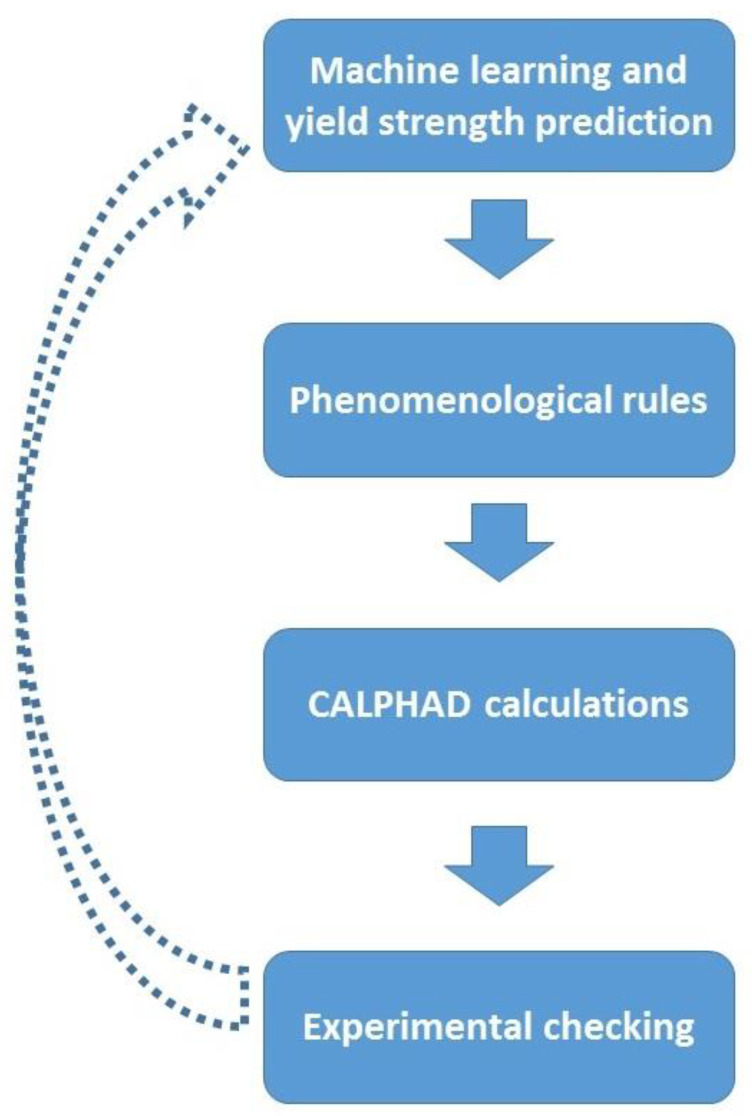
Schematic representation of the algorithm of model alloy selection.

The algorithm for the model alloy selection is shown in Figure 1. At the first stage, the composition space area of Al-Cr-Nb-Ti-V-Zr system alloys was selected. The size of this area is defined by the maximum and minimum concentrations of the components. The concentration range was not limited by equatomic composition and was increased to the interval of 0–50 at.% for Nb, Ti, V and Zr and 0–15 at.% for Al and Cr. The lower concentrations for Al and Cr were used to avoid the formation of intermetallic phases and/or ordering of the matrix phase [24,55]. Since Al and Cr had a narrower concentration range, a 1% step of concentration change was used; for other elements the step was 5%. The total number of potential alloys was therefore 29,269.

#### 2.1.1. Machine Learning

The yield strength of metallic materials can be either measured directly or predicted; the physically based prediction however is usually based on rather complicated and long calculations [63,64]. In the case of thousands of alloys, the use a surrogate (approximate) model is more reasonable. This model is trained on a dataset that includes known values of the calculated characteristic and a set of corresponding features. A trained surrogate model can predict the values for a characteristic set of alloys which were not used for training. In comparison with the strict calculation, the accuracy of this approach is usually lower, but the procedure is significantly easier. In this work the machine learning approach was used for creating a surrogate model for the prediction of the yield strength.

Meanwhile the accuracy of the surrogate model strongly depends on the dataset, the set of features, and the machine learning algorithm. Since our model focuses on the Al-Cr-Nb-Ti-V-Zr system, the dataset included only those alloys, which consisted of these elements [12,51,55,62]. The dataset sizes for room temperature, 600 °C and 800 °C were 30, 35 and 33 alloys, respectively. The datasets did not include data for those alloys which fractures in the elastic strain range, that is why the dataset for room temperature was the smallest. The set of features (δ, VEC, ΔH_mix, etc._; Table 1) was chosen based on an analysis of the literature. These features are related to their intrinsic properties which influence the formation of a solid solution, amorphous phase and/or intermetallic compound in HEAs, and affect the final yield strength [16,39,41,65,66,67].

**Figure 2:**
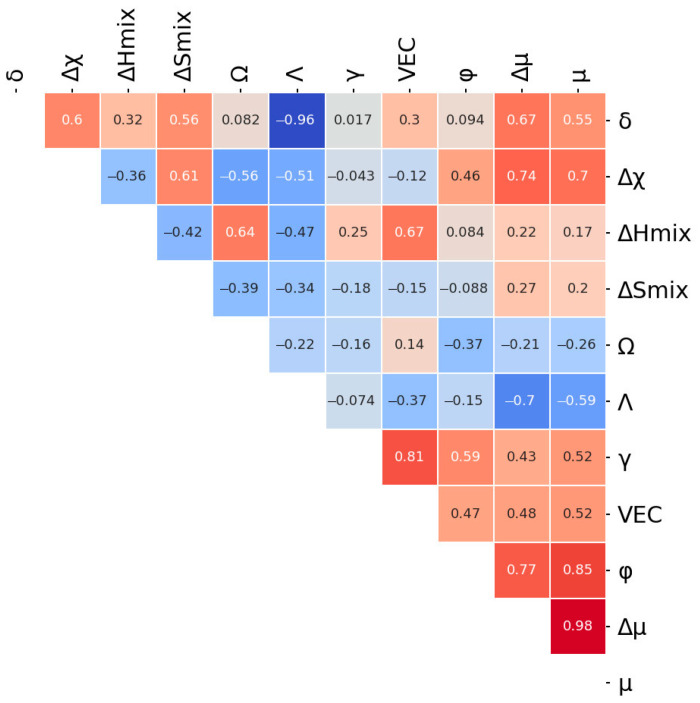
Pearson correlation coefficient map between different features.

In order to reduce the computer time and to improve the surrogate prediction model efficiency, a correlation analysis was used to remove unnecessary features. A Pearson correlation coefficient map between different features was constructed (Figure 2). The correlation coefficient is calculated as follows:

$$r=\frac{\sum \left(x-m_{x}\right)\left(y-m_{y}\right)}{\sqrt{\sum {\left(x-m_{x}\right)}^{2}\sum {\left(y-m_{y}\right)}^{2}}}$$

where $m_{x}$ is the mean of the vector x and $m_{y}$ is the mean of the vector *y*. Each pair of the features with a correlation coefficient greater than 0.95 were considered as a highly correlated combination and one of the features was excluded from the model. The correlations of δ–Λ and μ–Δμ were found to be more 0.95, therefore the δ and μ were omitted.

The choice of the optimal machine learning algorithm included a few stages. Firstly, seven well-known machine learning algorithms [68], such as a ridge regression algorithm (rid), support vector regressions with a linear kernel (svr.lin), a polynomial kernel (svr.poly), and a radial basis function kernel (svr.rbf), a regression tree algorithm (tree) and a k-nearest neighbor algorithm (knn) were compared. Each algorithm, in addition to a set of training data, includes also its own parameters (hyperparameters) so that the prediction accuracy can vary depending on the value of the hyperparameters as well. Grid search with root mean square error estimation were used for selecting optimal values of the hyperparameters for each algorithm.

**Figure 3:**
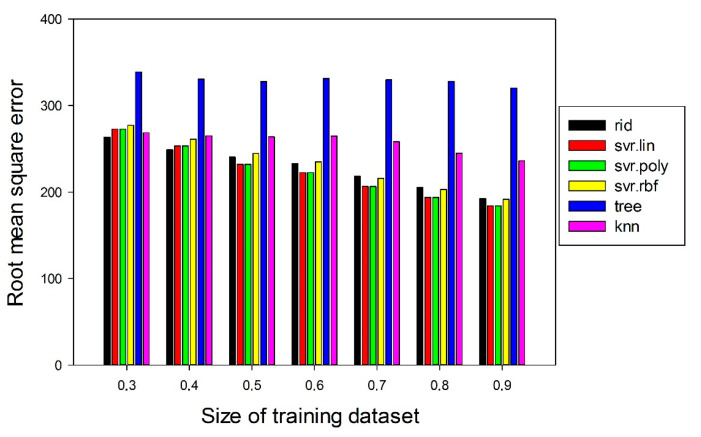
Dependence of root mean square error on training dataset size (fraction of the whole initial dataset) for different machine learning algorithms.

To calculate the prediction accuracy, the obtained initial dataset was split into a training dataset and a testing dataset. Since the size of the initial dataset was rather small (30–35 alloys, depending on temperature), it was important to choose the optimal ratio of the new-forming training and test datasets in all used algorithms to attain the best accuracy of the prediction. To this end, the size of the training datasets was varied in an interval of 0.3–0.9 of the full dataset. The surrogate models were trained using the training dataset, and then the models were used for the prediction of the yield strengths of the testing set alloys and for the calculation of the root mean square error. The diagram showing the root mean square error as a function of the training dataset size for all algorithms used for the yield strength prediction is shown in Figure 3. The error slightly depends on the training dataset size for two algorithms—tree and knn, For other algorithms the error decreases with an increase in the size of the training set. The optimal size of the training dataset was defined at 0.7 of the whole initial datasets.

**Figure 4:**
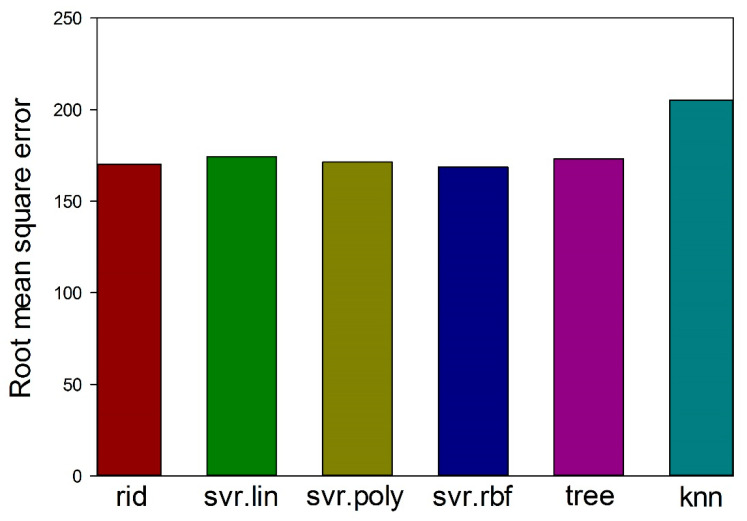
Ratio of root mean square errors for the models calculated using 50 bootstrap datasets.

After that, the most efficient algorithm was determined using a well-known in statistic approach bootstrap with replacement [68]. A total of 50 bootstrap datasets with a size of 0.7 of the initial datasets, each created by choosing random alloys from the initial dataset, and the alloys in the bootstrap datasets can be used more than once (even in one bootstrap datasets). These bootstrap datasets were used for training the algorithms to predict all the data points in the initial dataset and to calculate the root mean square error for all the machine learning algorithms. Among two algorithms showing the minimum prediction error (svr.rbf and rid; Figure 4), svr.rbf was trained and used for the prediction of the yield strength of HEAs at room at 20 °C, 600 °C or 800 °C. A cross-validation approach was also used for pretesting of the svr.rbf algorithm.

**Figure 5:**
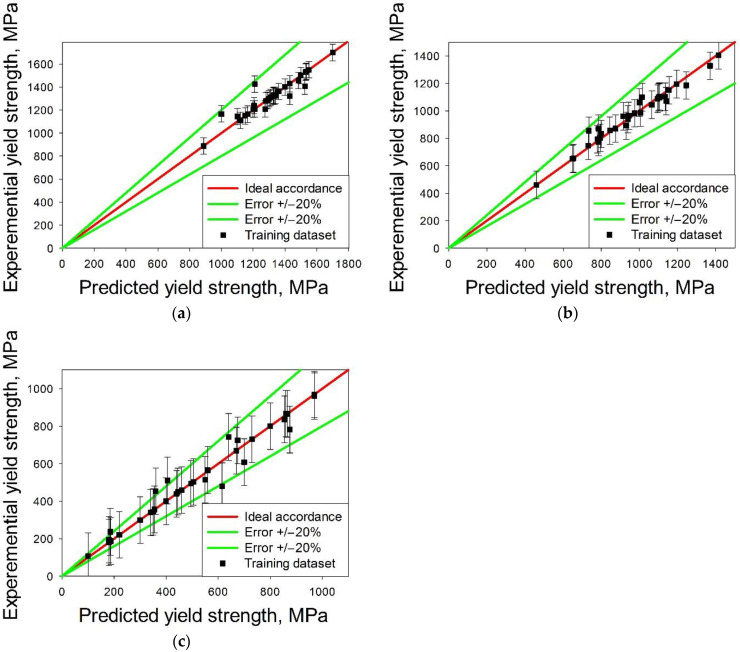
Experimental and predicted values of yield strength at 20 °C (**a**), 600 °C (**b**) and 800 °C (**c**) for training dataset. The predicted values were obtained using the svr.rbf surrogate model.

Figure 5 shows the comparison of the predicted and experimental values of the yield strengths at 20 °C, 600 °C or 800 °C; the predicted values were obtained using the svr.rbf surrogate model. One can see that the prediction for room temperature was more accurate than that for high temperatures. The bagging (from bootstrap aggregating) [68] approach was used for improving the prediction accuracy. To this end, 1000 datasets with a size of 0.7 of the initial datasets were randomly selected for (i) training surrogate model, (ii) yield strength prediction and (iii) calculating the average yield strength. The alloys with the yield strength less than the average value were excluded from consideration, due to which the number of the alloys was reduced to 11770. Then, the phenomenological phase formation criteria and the CALPHAD approach were used to select predominantly single-phase alloys.

#### 2.1.2. Phenomenological Rules

At first phenomenological models for the phase formation in HEAs were used to select single-phase alloys. The advantages and limitations of these models were thoroughly discussed elsewhere [25]. The general purpose of this step was to reduce the computing time, since the calculation using CALPHAD is much longer in comparison with that using the phenomenological models. The alloys were taken as single-phase ones if *δ* < 5.4%, VEC < 6.87, −16.25 kJ/mole ≤ ∆*H_mix* ≤ 5 kJ/mole, Ω > 1.1, *φ* > 7 and *η* > 0.19 [69]. The equations used for the calculations are presented below and in Table 1:

(1) $$\varphi =\frac{S_{mix}-\frac{\left|\Delta H_{mix}\right|}{T}}{S_{over}}$$

(2) $$\eta =\frac{TS_{config}}{H_{ij}^{IM}}$$

After calculations, 1250 candidate alloys with a presumably single-phase structure were selected for further consideration.

#### 2.1.3. CALPHAD Calculations

At the last stage, densities of the 1250 potential alloys were calculated using the rule of mixtures. Then, specific yield strength for each potential alloy was calculated as the ratio between the predicted yield strength and the calculated density. A total of 80 alloys with the highest sum of the specific yield strength at 20 °C, 600 °C and 800 °C were chosen. Their phase compositions were calculated using a Thermo-Calc (version 2020a) and a TCHEA3 database (High Entropy Alloys version 3.1) for 1200 °C since this temperature is usually used for homogenization annealing of Al-Cr-Nb-Ti-V-Zr RHEAs [51,52,53,54,55,56,57,58,59,60,61,62]. As a result, six model alloys were selected (Table 2). Six of them possessed the greatest sum of specific yield strength and they were either single-phase or contained less than 10% of a second phase(s). Another two alloys (A3 and A4) were chosen based on only high specific yield strength values irrespective of the phase composition. The experimental values of the yield strength of the model alloys were used for the prediction of computational accuracy. Additionally, the obtained experimental values of strength were included in the dataset for the development of the next generation of new alloys.

### 2.2. Experiment

The model alloys were produced by vacuum arc melting, using proper mixtures of pure metals with purities of better than 99.9 wt.%, in a Ti-gettered argon atmosphere. The measured compositions of the model alloys are listed in Table 3. The alloys were remelted five times to improve their homogeneity. The obtained ingots were then sealed in vacuumed (10^−2^ torr) quartz tubes and soaked at 1200 °C for 10 h. After the homogenization annealing, samples for compression test and microstructure investigation were cut out using an electric discharge machine.

The phase composition was studied using X-ray diffraction (XRD) on a RIGAKU diffractometer with CuKα radiation. SEM investigations were carried out using either FEI Quanta 600 FEG or Nova NanoSEM microscopes; both instruments were equipped with back-scattered electron (BSE) and energy-dispersive X-ray spectroscopy (EDS) detectors. Specimens for structural investigations were finished with OP-S suspension (the abrasive particle size of 0.04 μm). The chemical composition of the alloys was measured using SEM-EDS with a scanning area of 2 × 2 mm^2^.

The microhardness was measured using a Wolpert group 402mvd microhardness tester. The load and dwell time were 300 g and 10 s, respectively. The microhardness value was averaged on five measurements. Rectangular specimens measured 8 × 5 × 5 mm^3^ were compressed using an Instron 300LX testing machine equipped with a radial heating furnace. The tests were carried out at 20 °C, 600 °C or 800 °C with an initial strain rate of 10^−4^ s^−1^ till 50% of height reduction (or till fracture).

## 3. Results

### 3.1. Machine Learning Prediction of Composition-Properties Relationships in Alloys of the Al-Cr-Nb-Ti-V-Zr System

**Figure 6:**
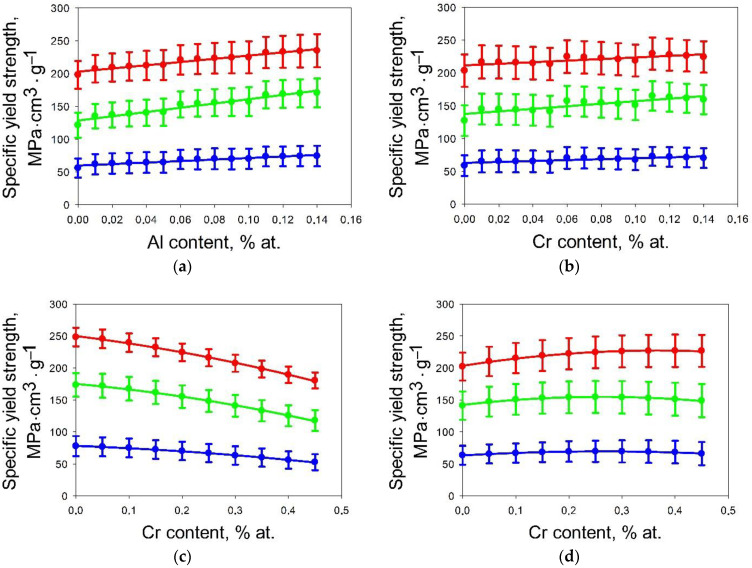

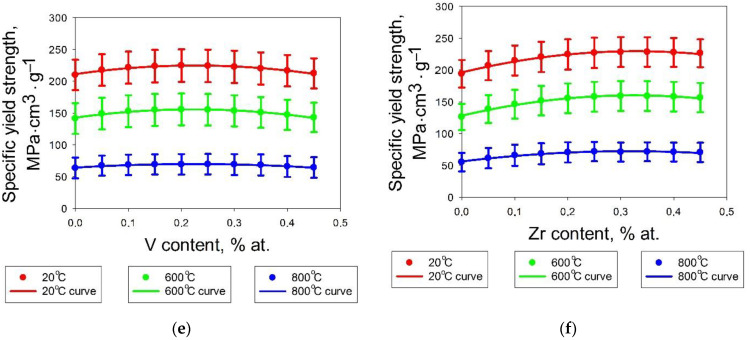
Dependence of mean specific yield strength on concentration of Al (**a**), Cr (**b**), Nb (**c**), Ti (**d**), V (**e**) and Zr (**f**).

The number of alloys with a fixed percentage of each certain element (while others vary in the intervals 0–15% for Al, Cr or 0–45% for Nb, Ti, V, Zr) can attain several thousand variants. To evaluate the main trends of the influence of each element on strength, the maximum, minimum and average values (upper, lower, and middle points of each error bar in Figure 6) of the specific yield strength of alloys at fixed values of the elements (Al, Cr, Nb, Ti, V or Zr) was analyzed. For titanium, vanadium, zirconium, and niobium a parabolic law was observed for the dependence of specific yield strength on the content of elements. An increase in the concentration of titanium (Figure 6d), vanadium (Figure 6e) and zirconium (Figure 6f) increases the specific yield strength to the maximum value with a subsequent decrease. An addition of niobium (Figure 6c) resulted in a continuous decrease in the specific yield strength. For Al (Figure 6a) and Cr (Figure 6b) within a narrower interval of concentration (0–15%) a linear increase in strength can be suggested.

**Figure 7:**
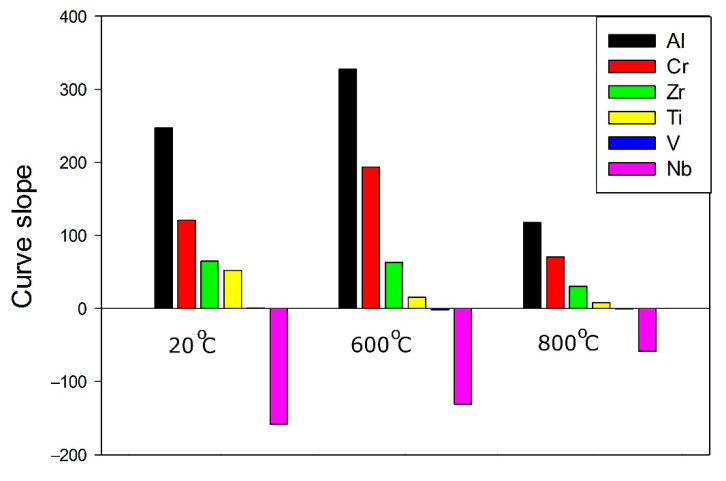
Slope of curves (see Figure 6) for all elements for concentrations less than 15% at 20 °C, 600 °C or 800 °C.

For all elements, the dependence of the specific yield strength on concentration (below 15%) can be extrapolated linearly with the Pearson’s coefficient of more than 0.85. For further insight into the effect of the elements (and their content) on the specific yield strength of the Al-Cr-Nb-Ti-V-Zr system alloys, the slope of the specific yield strength-concentration curves in the interval 0–15% was analyzed. Figure 7 shows that the slopes for Al, Cr and Zr are higher than those for other elements at all investigated temperatures. Thus, the contents of Al, Cr and Zr have the greatest influence on the specific yield strength, while the effect of V and Ti was lower; the addition of Nb have a negative effect on the specific yield strength. Chromium and zirconium have the largest and smallest atomic radii (129 and 160 picometers) and it can be assumed that these elements make the main contribution to hardening due to solid solution hardening. In this case, the atomic radius of aluminium is close to the rest of the elements (143 picometers) and its contribution to solid solution hardening is not as great as for chromium and zirconium.

In this case, aluminium can contribute to the ordering of alloys and the formation of intermetallic compounds, and it can be assumed that its main contribution to strengthening is associated with the formation of secondary phases. For Al and Cr the maximum yield strength was observed at concentrations of 14% in each case. At the same time, the maximum values of specific yield strength at room temperature corresponded to 40% of Nb, 20% of V and 35% of Zr. Based on these concentrations, perspective areas of the composition space of Al-Cr-Nb-Ti-V-Zr high-entropy alloys were selected. For all the selected model alloys, the concentration of at least one element corresponded to the maximum strength.

In the A1 (Al_14_Cr_1_Nb_10_Ti_45_V_25_Zr_5_) and A4 (Al_13_Cr_7_Nb_5_Ti_45_V_15_Zr_15_) alloys the maximum strength at room temperature was supposed to be caused by the appropriate concentrations of Ti and Al. In the A3 (Al_14_Cr_11_Nb_5_Ti_35_V_25_Zr_10_) and A5 (Al_13_Cr_12_Nb_20_Ti_20_V_35_) alloys the maximum strength thought to be attained due to the presence of V, Al and Cr; in A6 (Al_13_Cr_2_Nb_20_Ti_30_V_25_Zr_10_) due to V and Al and in A2 (Al_10_Nb_20_Ti_35_V_15_Zr_20_) due to Ti.

### 3.2. Comparison between the Predicted and Actual Structure of Al-Cr-Nb-Ti-V-Zr Alloys

**Figure 8:**
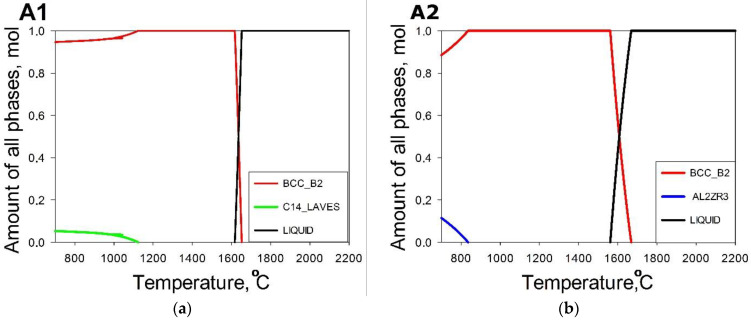

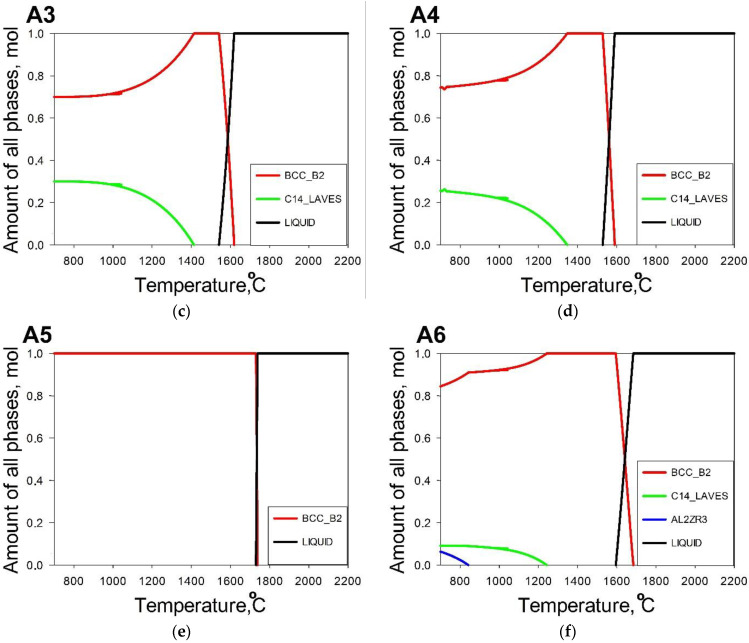
Calculated phase diagrams for the model alloys A1 (**a**), A2 (**b**), A3 (**c**), A4 (**d**), A5 (**e**) and A6 (**f**).

Phase diagrams for the model alloys obtained using the CALPHAD approach (Thermo-Calc software) are shown in Figure 8. All the program alloys crystallize through a single bcc phase field. However, at 1200 °C (heat treatment temperature, used in the current study) only A1and A5 alloys have a single bcc phase structure. In the rest of the alloys, a secondary hexagonal (C14) Laves phase was expected to appear between the solidus temperature and 1200 °C. The fraction of the Laves phase was evaluated to be <0.1 in A6, and ~0.13 and 0.21 in the A4 and A3 alloys, respectively. The fraction of the Laves phase gradually increased with a decrease in temperature for most of the alloys, with the exception of the A5 alloy which retains the single bcc phase structure down to 700 °C and A2 alloy, where some amount of Zr_3_Al_2_ phase formed at T < 840 °C.

**Figure 9:**
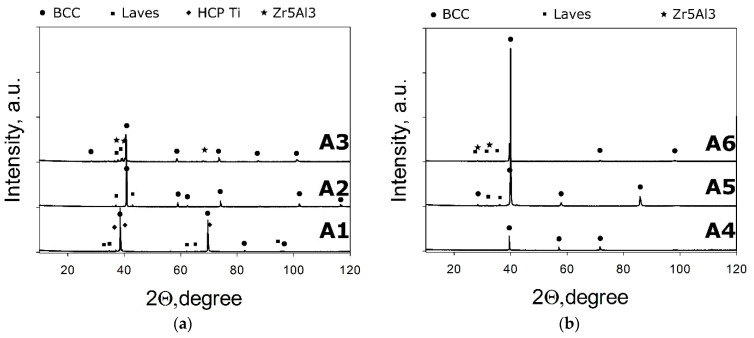
XRD patterns of the A1–A3 (**a**) and A4–A6 (**b**) model alloys.

XRD analysis (Figure 9) suggests that the A4 alloys comprise of the bcc phase only. The A5 and A2 alloys have additional tiny peaks due to the presence of the Laves phases. Other model alloys contain, in addition to the bcc phase, more than one phase (Laves and hcp phases in A1; Laves and Zr_5_Al_3_ in both A3 and A6). The presence of the C14 Laves phase in the A1, A3 and A6 alloys agrees with the CALPHAD calculations (Figure 9). However, CALPHAD did not predict the single-phase condition in the A4 alloy as well as the appearance of the Zr_5_Al_3_ phase in A3. Some discrepancies between CALPHAD-based predictions and experimental results are well-documented [38,70] and therefore are not surprising. However, the CALPHAD approach gives quite reliable qualitative data, particularly in combination with other prediction methods, and therefore can be used for the assessment of expected phase compositions in developed alloys.

**Figure 10:**
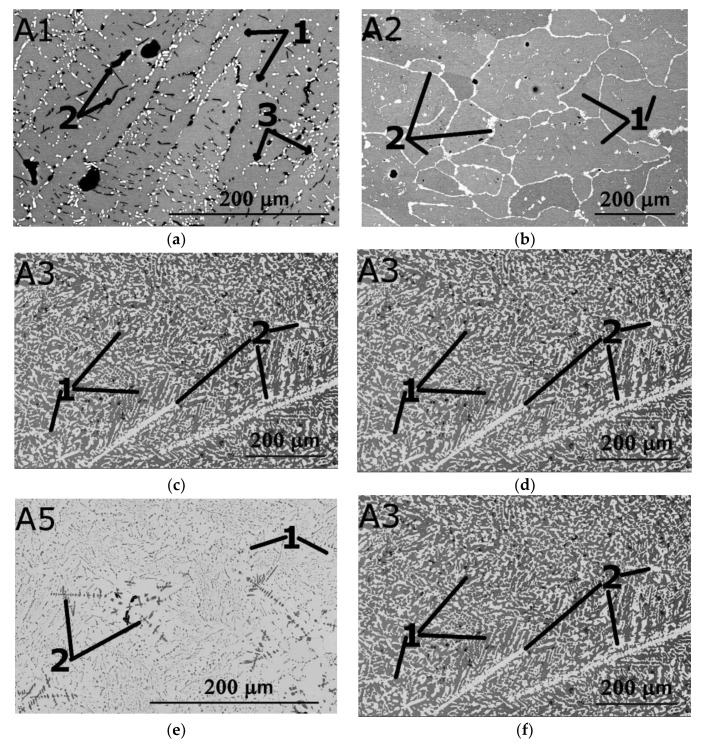
SEM-BSE images of the A1–A6 alloys, A1 (**a**), A2 (**b**), A3 (**c**), A4 (**d**), A5 (**e**) and A6 (**f**).

Microstructures of the model alloys are shown in Figure 10. SEM images of all alloys demonstrate multiphase structures. Microstructures of the alloys A2 and A4 consist of grains ~100–200 μm in size with second phase(s) precipitations located mainly along grain boundaries. In the A4 and A2 alloys the second phase particles create a continuous intergranular layer with the thickness from 0.6 μm (in A4) to 4.8 μm (in A2). The volume fraction of the secondary phases was ~1% in A4 and ~27% in A2. The alloys A1, A3, A5 and A6 rather have a dendritic microstructure with second phase(s) located in the interdendritic areas. In the A1, A3, and A5 alloys, the second phase(s) are mostly presented as separate particles while in A6 the second phase creates a continuous network. The volume fraction of the second phase(s) was ~6% in A1, 41% in A3, 10% in A5 and 7% in A6. Chemical compositions of the phases in the model alloys are shown in Table 3; a more detailed investigation of microstructures was out of the scope of the present work.

For four of the six model alloys, the amount of the second phase(s) was less than 10%. However, differences between the measured phase compositions (Table 3) and CALPHAD calculations were more pronounced. For the alloys A4 and A5, the content of the second phase was small and therefore the peaks of the second phase was not observed in the X-ray diffraction pattern. For A2 the calculated phase composition corresponded to the actual one only qualitatively. Some discrepancies between the calculated phase composition and the actual one was observed in A3. Meanwhile, for the A1 alloy, one can notice the presence of a phase that was not calculated in the CALPHAD calculation.

### 3.3. Comparison of Predicted and Measured Mechanical Properties of the Al-Cr-Nb-Ti -V-Zr Alloys

The maximum and the minimum values of microhardness was observed in the A3 alloy (650 HV) and A6 alloy (489 HV), respectively (Table 4). Four alloys (A1, A4, A5 and A2) have the microhardness values in a narrow interval 540–556 HV.

**Figure 11:**
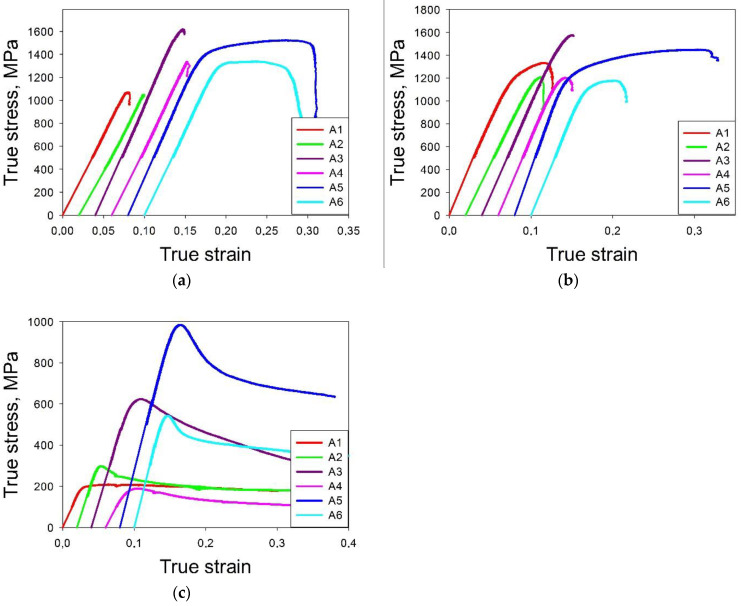
Compression true stress–true strain curves for model alloys at 20 °C (**a**), 600 °C (**b**) and 800 °C (**c**).

Compression stress–strength curves of the model alloys at 20 °C, 600 °C and 800 °C are shown in Figure 11. The measured and predicted values of the mechanical properties are listed in Table 4. The yield strengths of the model alloys at 20 °C are in a range from 1049 MPa for the A2 alloy to 1608 MPa for the A3 alloy; the later showed the maximum microhardness as well. The majority of the model alloys has the yield strengths at room temperature is around 1300 MPa, however. Ductility over 1% was observed in the A5 and A6 alloys (16.6 and 13.8, respectively); A4 showed ~ 1% ductility. The A1 and A2 alloys fractured in the elastic region; for these specimens the (yield) strength values were evaluated using microhardness tests. The ratio between the microhardness and yield strengths was estimated using the corresponding values for more ductile alloys (i.e., A3, A4, A5 and A6). This ratio was found to be 2.38, therefore the estimated strength values for the A1 and A2 alloys can be adopted as 1316 and 1323 MPa, respectively.

At 600 °C, the highest and the lowest values of the yield strengths were shown by the A3 and A6 alloys (1385 and 1048 MPa, respectively), similar to 20 °C. The yield strengths of other model alloys were around 1100 MPa. All alloys showed some ductility (i.e., did not fracture in the elastic region), yet only the A1 and A5 alloys had a ductility over 5% (17.2% and 5.5%, respectively). At 800 °C, all the model alloys did not fracture till 50% height reduction. Only three model alloys (A3, A5 and A6) showed yield strength more than 300 MPa (556, 898 and 509 MPa, respectively). For other alloys, the yield strengths were in a range between 152 and 287 MPa. The best strength/ductility ratio at all tested temperatures was demonstrated by the A5 alloy. Comparison of this alloy with 47 various RHEAs of the Al-Cr-Nb-Ti-V-Zr system and equiatomic four-, five- and six-components alloys of the Nb-Ti-V-Zr-Mo-Ta-Hf-W system collected in [14] have shown that the yield strength/density ratio of the A5 alloy at 800 °C is one of the highest. Only two alloys ((AlCr_2_NbTiV and Al_0.5_CrNbTiVZr) have comparable density (5.95 and 6.23 g/cm^3^, respectively) and higher values of the yield strength (970 MPa in both cases). Other alloys possess lower either strength of density (or both).

**Figure 12:**
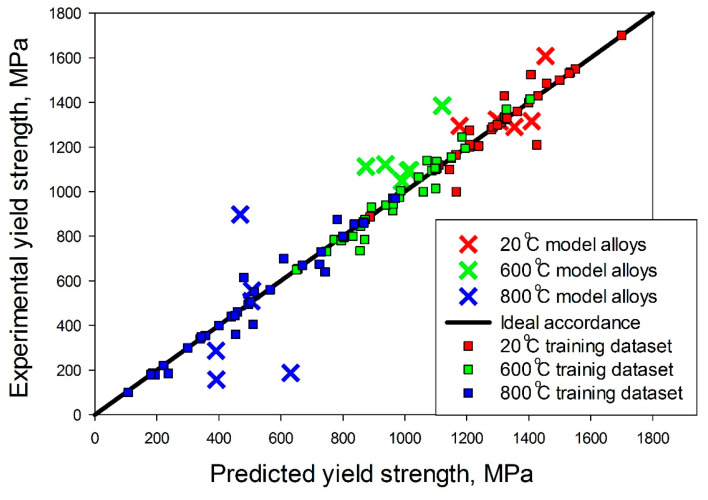
Predicted and experimental values of yield strength at 20 °C, 600 °C and 800 °C.

The experimental and predicted by machine learning method yield strength values are shown in Table 4 and Figure 12. The surrogate model results were in good prediction accuracy at 20 °C and 600 °C; at 800 °C the prediction error was more pronounced. The mean prediction error is 7% at 20 °C and 12% at 600 °C, which is comparable to the accuracy of such predictive systems. Accuracy of prediction hardness for Al-Co-Cr-Cu-Fe-Ni system near 80% [47,71]. Li et al. [49] had a mean error between molecular dynamic simulation of tensile and strength predicted by machine learning less than 2%. At 800 °C, the surrogate model showed the prediction error less than 20% for only two model alloys. While in work [50] for high-entropy alloys MoNbTaTiW and HfMoNbTaTiZr at 800 °C, the prediction accuracy is 95%. Thus, our proposed model for predicting the yield stress has good accuracy for room temperature and 600 °C, but for higher temperatures its accuracy is insufficient. In this work, only alloys of the Al-Cr-Nb-Ti-V-Zr system were used to train the surrogate model. An increase in the sample due to the inclusion of alloys of the system Al-Cr-Nb-Ti-V-Zr-Mo-Ta-Hf-W does not lead to an increase in accuracy, but to a slight decrease (0.5% for 20 °C, 3% for 600 °C, for 800 °C the accuracy decreases by one and a half times). However, expanding the training dataset to include newfound alloys will improve the prediction accuracy. When the six model alloys obtained in this work are included in the dataset, the standard deviation for 20 °C decreases by 20% (from 145 to 116), for 600 °C by 6% (from 161 to 151); for 800 °C, the increase in the sample did not affect the standard deviation.

One of the factors that influenced the relatively low prediction accuracy at 800 °C, can be associated with different melting temperatures of the alloys in the training dataset. Therefore, 800 °C for different alloys corresponds to different homological temperatures. However, 800 °C corresponds to 0.35–0.43 of the melting temperature (T_m_) for the alloys from the training dataset, and (0.38–0.41) T_m_ for the model alloys. This difference does not seem too high to cause such a low prediction accuracy. Another factor, associated with a transition from an athermal plateau to strong temperature dependence in bcc metals, seems more important. This transition was observed in high-entropy alloys with a bcc lattice, as well as in conventional bcc metals and alloys at temperatures of about (0.4–0.5) T_m_. This means that for some alloys from the training dataset areas, the athermal plateau can be observed at 800 °C, while other alloys demonstrate a strong temperature dependence. This heterogeneity in the training dataset may result in a severe spread in the yield strength values, thereby decreasing the prediction accuracy at 800 °C.

The obtained results suggest that the strength and phase composition of high-entropy alloys can be rather successfully predicted using a combination of various approaches: machine learning, phenomenological rules and CALPHAD modeling. The proposed approach obviously requires further improvement through the involvement of additional models and computing methods. Prospective work should focus on (i) an improvement of the prediction accuracy, especially at high temperatures and (ii) an expansion of the predicted characteristics (e.g., ductility, oxidation resistance, etc.).

## 4. Conclusions

This section is not mandatory but can be added to the manuscript if the discussion is unusually long or complex.

A combined approach, including phenomenological rules, CALPHAD and machine learning, was used in the search for alloys with desirable properties (phase composition and yield strength). As a result, the following conclusions were made:

1. The use of a combination of CALPHAD and phenomenological rules does not result in an accurate prediction of the phase composition of the alloys; only one of them had a desirable single-phase structure. However, in four model alloys the second phase(s) did not exceed 10%, thereby suggesting the good potential of this approach for the selection of alloys with a desirable phase composition.
2. The surrogate model based on a support-vector machine algorithm for the prediction of the yield strength showed good accuracy at 20 °C and 600 °C (the error of prediction was less than 20% for all alloys except one). However, at 800 °C, the error of prediction was worse than 20% for only two model alloys. Relatively low prediction accuracy at 800 °C can be associated with the proximity of this temperature to the transition point between the athermal plateau and the strong temperature dependence in bcc alloys, causing, in turn, a severe spread in the yield strength of the training dataset alloys.
3. For the Al-Cr-Nb-Ti-V-Zr system, the content of aluminum, chromium and zirconium have the greatest influence on the specific yield strength. The effect of vanadium and titanium is lower; an addition of niobium has a negative effect on specific yield strength.
4. One of the predicted alloys (A5: Al_13_Cr_12_Nb_20_Ti_20_V_35_) possesses an excellent combination of strength (1295 MPa at 20 °C, 1113 MPa at 600 °C and 898 MPa at 800 °C) and ductility (16.8% at 20 °C, 5.5% at 600 °C and >50% at 800 °C) in the interval 20–800 °C.

## Figures and Tables

**Table 1:** List of input features for the surrogate prediction model.

Feature	Equation for Feature Calculation
The difference in atomic radii between elements (δ)	$\delta =\sqrt{\sum c_{i}{\left(1-\frac{r_{i}}{\bar{r}}\right)}^{2}}$, $\bar{r}=\sum c_{i}r_{i}$
Valence electron concentration $\left(\mathrm{VEC}\right)$	$\mathrm{VEC}=\sum c_{i}{\left(\mathrm{VEC}\right)}_{i}$
Enthalpy of mixing ($\Delta H_{mix}$)	$\Delta H_{mix}=\sum \sum 4\Delta H_{ij}^{mix}c_{i}c_{j}$
Difference in electronegativity between elements	$\Delta \chi =\sqrt{\sum c_{i}{\left(\chi_{i}-\sum c_{j}\chi_{j}\right)}^{2}}$
Configurational entropy	$\Delta S_{mix}=-R\sum c_{i}\ln\left(c_{i}\right)$
Work function	$\phi =\sum c_{i}\phi_{i}$
Shear modulus	$\mu =\sum c_{i}\mu_{i}$
Difference in shear modulus	$\Delta \mu =\sum \frac{2\cdot \left(\mu_{i}-\mu \right)}{\mu_{i}+\mu }$
γ parameter	γ=1−rs+r¯2−r¯2rs+r¯21−rl+r¯2−r¯2rl+r¯2
Λ parameter	$\Lambda =\frac{\Delta S_{mix}}{\delta^{2}}$
Ω parameter	$\Omega =\frac{T_{m}\Delta S_{mix}}{\left[\Delta H_{mix}\right]}T_{m}=\sum c_{i}T_{i}$

**Table 2:** Chemical compositions of the model alloys.

Alloy	Content, at.%					
	Al	Cr	Nb	Ti	V	Zr
A1	14	1	10	45	25	5
A2	10	-	20	35	15	20
A3	14	11	5	35	25	10
A4	13	7	5	45	15	15
A5	13	12	20	20	35	-
A6	13	2	20	30	25	10

**Table 3:** The chemical compositions of model alloys (in at. %).

Alloys		Al	Cr	Nb	Ti	V	Zr
A1	Nominal composition	14	1	10	45	25	5
	Actual chemical composition	13	0.1	10.1	43.8	27.8	5.2
	1 (matrix)	15.1	0.1	10.7	43.1	27.4	3.6
	2 (dark particles)	2.2	0	3.9	78.6	4.2	11.1
	3 (light particles)	16.9	0.4	11.2	39.1	25.2	7.2
A2	Nominal composition	10	-	20	35	15	20
	Actual chemical composition	9.6	0	20.4	35.5	13.3	21.2
	1 (matrix)	10	0	21.3	35.6	12.8	20.3
	2 (light phase)	11	0	20.2	33	12.9	22.9
A3	Nominal composition	14	11	5	35	25	10
	Actual chemical composition	15.5	11.3	6.2	36.2	20.3	10.5
	1 (grey)	13.8	11.1	7.2	39.2	24.3	4.4
	2 (light)	15.5	10.8	4.8	32.7	15	21.2
A4	Nominal composition	13	7	5	45	15	15
	Actual chemical composition	11.3	6.8	7.3	49.6	14.5	10.5
	1 (matrix)	12.6	6.7	7.3	49	14.3	10.1
A5	Nominal composition	13	12	20	20	35	-
	Actual chemical composition	14.1	13.5	23.3	22.5	25.2	1.4
	1 (matrix)	15	15.3	26.1	14.8	28.3	0.5
	2 (dark particles)	3.1	3.3	7	78.3	7.5	0.8
A6	Nominal composition	13	2	20	30	25	10
	Actual chemical composition	12	1.2	22.2	30.9	19.5	14.2
	1 (matrix)	13.1	1.6	23.5	31.6	22.3	7.9
	2 (light phase)	14.2	1.9	20.5	29.1	20.1	14.2

**Table 4:** Measured and predicted yield strength for 20 °C, 600 °C and 800 °C and microhardness of the model alloys

Alloy	Microhardness, HV	Yield Strength, MPa						
		20 °C			600 °C		800 °C	
		Measured	Estimated Using Microhardness	Predicted	Measured	Predicted	Measured	Predicted
A1	553	1070 *	1316	1409	1093	1011	187	631
A2	556	1049 *	1323	1297	1122	937	287	390
A3	650	1608		1454	1385	1120	556	506
A4	552	1337		1306	1096	1016	157	392
A5	540	1295		1177	1113	874	898	468
A6	489	1290		1353	1048	991	509	504

* The maximum strength attained before fraction in the elastic region.

## Data Availability

The data presented in this study are available on request from the corresponding author.
